# The impact of referral letter quality on timely access to specialised mental health care: a quantitative study of the reliability of patient triage

**DOI:** 10.1186/s12913-022-08139-3

**Published:** 2022-06-02

**Authors:** Marit Nymoen, Eva Biringer, Øystein Hetlevik, Olav Thorsen, Jörg Assmus, Miriam Hartveit

**Affiliations:** 1 Department of Research and Innovation, Helse Fonna Health Trust, Haugesund, Norway; 2 Department of Global Public Health and Primary Care, University of Bergen, Bergen, Norway; 3 The General Practice and Care Coordination Research Group, Stavanger University Hospital, Stavanger, Norway; 4 Center for Clinical Research, Haukeland University Hospital, Bergen, Norway

**Keywords:** Patient triage, Needs assessment, Mental health services, Referral and consultation, Health priorities, Hospital referrals

## Abstract

**Background:**

Patients referred to specialised mental health care are usually triaged based on referral information provided by general practitioners. However, knowledge about this system’s ability to ensure timely access to and equity in specialised mental health care is limited. We aimed to investigate to the degree to which patient triage, based on referral letter information, corresponds to triage based on a hospital specialist’s consultation with the patient, and whether the degree of correspondence is affected by the quality of the referral letter.

**Methods:**

We gathered information from three specialised mental health centres in Norway regarding patients that were referred and offered health care (*N* = 264). Data consisted of triage decisions for each patient (i.e., the hospital specialist’s assessment of maximum acceptable waiting time), which were determined on the basis of a) referral information and b) meeting the patient. Referral letter quality was evaluated using the Quality of Referral information-Mental Health checklist. The reliability of priority setting and the impact of referral letter quality on this measure were investigated using descriptive analyses, binary logistic regression and Nadaraya-Watson kernel regression.

**Results:**

In 143 (54%) cases, the triage decision based on referral information corresponded with the decision based on patient consultation. In 70 (27%) cases, the urgency of need for treatment was underestimated when based on referral information compared with that based on information from patient consultation. Referral letter quality could not explain the differences between the two triage decisions. However, when a cut-off value of 7 on the Quality of Referral information-Mental Health scale was used, low-quality letters were found more frequently among patients whose urgency of need was underestimated, compared with those whose need was overestimated.

**Conclusions:**

Deciding the urgency of patient need for specialised mental health care based on referral information is a reliable system in many situations. However, the possibility of under- and overestimation is present, implying risks to patient safety and inappropriate use of resources. Improving the content of referral letters does not appear to reduce this risk when the letters are of acceptable quality.

**Trial registration:**

NCT01374035.

## Background

Managing access and triaging patients by the urgency of their health care needs is necessary because of the large, and in many cases increasing, demand for specialised mental health care services [1, 2]. A consistent and reliable triage system can promote timely and equitable health care, which is often essential for patient outcomes [2–4]. However, estimating the urgency of treatment need for referred patients can be challenging, particularly within mental health care [2, 3]. Underestimation of the urgency of treatment needs implies risks to patient safety, including the risk of self-harm, suicide and negative impact on prognosis [4, 5]. Overestimation (i.e., giving priority to patients with less urgent needs) implies a risk of inappropriate utilisation of scarce resources. Different systems to aid decision-making have been developed in various countries and health care systems [3, 6]. Integrative measures, such as digital consensus meetings [7], and “open door” systems in which patients decide themselves when to be admitted [8], have been tested. However, many countries, including Australia, the United Kingdom and France, manage access to specialist health care using a referral system [6]. Patients are referred by general practitioners (GPs) or other primary health care services to specialised health care, and triaged by information provided in the referral letter [3, 6]. Referral letter quality is often assumed to be an important factor in the limited reliability of the referral triage system, thereby constituting a risk to timely access to care [2, 9].

Literature on the reliability of the referral triage system within mental health care is scarce. Holman and colleagues compared the triage decisions of several specialist mental health care teams using the same 20 vignettes of referral letters [2]. The results revealed low agreement between teams, indicating a risk of both under- and overestimation of the urgency of need for treatment [2]. Standardising triage decisions has been deemed necessary for correct assessment of treatment need [10]. Context-specific tools have been developed for assessing the urgency of treatment need to improve patient prioritisation [11]. A recent review of patient prioritisation tools for elective care indicated that 50% of the prioritisation tools that had been tested for reliability were categorised as acceptable to good [11].

The current study sought to investigate the reliability of specialists’ assessment of patients’ urgency of need for specialised mental health care, and to investigate whether the quality of referral letters can explain this reliability. Specifically, we aimed to examine:

1. The degree to which triage decisions based on referral letter information correspond with triage decisions based on consultation with the patient.
2. Whether the quality of referral letters can explain differences in triage decisions.

## Methods and design

This quantitative study included a naturalistic sample of patients referred from general practice to specialist mental health care in Norway. In addition to collecting information about the triage decisions made in the existing referral assessment system in specialised mental health care, we asked mental health specialists to perform a similar triage, but based on information from meeting the patient. We compared the two triage decisions for each referral to investigate the reliability of the existing system of managing timely access to care for patients referred to specialist mental health care. In addition, we investigated the associations between the quality of referral letters and differences between the two triage decisions. The sampling was conducted before the outbreak of COVID-19, during autumn/winter 2019.

### Context

The study was conducted in Norway, in which the majority of specialised mental health care services are public and can be accessed by a maximum fee of approximately €250 per year [12]. Priority is determined by mental health specialists based on the information provided in the referral letter by a medical doctor in primary care [13]. Priority setting is regulated by law and national guidelines, using the severity of illness, expected impact of health care on quality of life and cost-utility as criteria [14, 15]. However, the regulations and guidelines for priority setting leave room for professional discretion [14, 15].

### Sample

A consecutive sample of patients referred to one of three specialist mental health care centres was included. These mental health centres are each responsible for a specific geographical area in one public health authority. Their catchment area vary from approximately 25,000 to 120,000 citizens. Otherwise, they are similar in their organisation and the health services they provide.

Rejected referrals were excluded. According to an estimate of statistical power (Spearman’s rho = 0.3 and power = 0.8), a sample of 84 included patients would yield sufficient statistical power for bivariate correlation tests. However, because of uncertainty in the power estimates, and to enable analysis at the sub-group level, we made an a priori decision to include a sample size of 250 patients.

For each referred patient, we gathered information about triage decisions based on referral letter information and triage decisions based on information from a patient consultation, and examined the referral letter to assess its quality. The dataset also included information regarding the patients’ sex, age group, diagnostic group, and whether the patient had received treatment from specialised mental health care in the last 5 years. The diagnosis group was defined by the diagnosis made after 6 months of treatment or at the end of treatment. The data were collected by medical secretaries at each mental health centre and did not include patient identification information.

### Reliability of triage decisions

In the Norwegian health system, all referrals are triaged by a hospital specialist. The triage is performed by deciding the maximum acceptable waiting time (in weeks) before starting the treatment, based on the information in the referral letter [13, 15]. National guidelines have been developed to aid the decision-making [15]. For the purposes of the present study, we introduced a second patient triage, in which the specialist used information from the first patient consultation, either via a direct meeting with the patient or via second-hand information from other health care professionals conducting the intake interview. This second triage is considered to be a “gold standard” in our approach, as it includes more information than a referral letter alone. In line with the aim of investigating the existing practice of giving priority to patients, we did nothing to hide information about the first triage decision for the specialist conducting the second triage. In some situations it was the same specialist conducting both triages. However, we encouraged the specialists assessing the urgency of patients’ needs the second time to do this regardless of the former triage decision.

Reliability was defined by the difference in triage decisions (i.e., the difference in the number of weeks between the two definitions of maximum acceptable waiting time). Because the patient risk of inappropriate triage is expected to be much higher when maximum acceptable waiting time is short, we introduced the following definition of reliable triage: When the triage decision based on patient consultation was between 0–5 weeks, the triage decision based on the referral information had to be equal. At 6–14 weeks we accepted a 1-week difference between each of the two prioritisations, and at more than 14 weeks a 2-week difference was accepted. Unreliable priority setting was divided into: a) overestimation, in which the triage based on patient consultation defined a higher maximum number of acceptable weeks to wait for treatment than the existing system based on referral information, and b) underestimation, in which the priority set after the patient consultation implied a shorter acceptable waiting time than the prioritisation based on referral letter information.

### Quality of referral letters

Referral letter quality was evaluated using the Quality of Referral information-Mental Health checklist (QRef-MH) developed by Hartveit and colleagues [9]. This checklist provides a compliance score regarding recommended content in referral letters to specialised mental health care, and includes information defined as important by mental health specialists, patient representatives and GPs [9]. QRef-MH scores have been found to have sound psychometrics properties, with moderate interrater reliability and substantial test–retest reliability [9]. The checklist consists of 19 items. The first three items concern patient identifiers and were therefore not included in the present dataset. The maximum score was 16. Items 4 to 19 in QRef-MH concern information about symptoms and level of functioning, previous or current illnesses and reasons for referral. Each item is scored “1” if the information is present, either explicitly confirming the information or stating that the information is not applicable. For example, the item regarding information about suicidality is scored “1” either if it is stated that the person is suicidal or if it states that they are not suicidal. An absence of information regarding suicidality in the referral letter is scored “0” [9].

The process of QRef-MH scoring was carried out in a stepwise fashion. Three researchers scored a random sample of 10 referral letters for an initial calibration between raters. After an additional 50 referrals, a second consensus meeting to detect any systematic errors was conducted. All referral letters were scored twice.

In addition to using the raw QRef-MH score, we dichotomized the scale to distinguish between low and medium or high quality. In the development of QRef-MH, almost all referral letters of low quality had a score of 7 or lower [9], which is why we used QRef-MH ≤ 7 vs. QRef-MH > 7.

### Analyses

Descriptive statistics were used to describe sample characteristics. Graphics (bubble plot) were used to display differences in triage decisions. The relationship between the deviation of triage decisions and QRef-MH score was investigated graphically for both the raw QRef-MH score and the appearance of QRef-MH > 7. Both representations were smoothed using a Nadaraya-Watson kernel regression [16, 17] with Gaussian kernel and bandwidths of 3 and 5, respectively. The predictive value of a QRef-MH score on the reliability of the referral triage, as well as age group, sex, whether the patient had received help from specialised mental health care in the last 5 years, if the hospital specialist usually partakes in triage decisions, and the number of weeks between priority assessments, was assessed using the logistic regression model with agreement between referral triage and the triage after patient consultation (yes/no) as dependent variable. We used a three-step-procedure: first, we estimated the univariate model for each predictor, and secondly we estimated the full model including all predictors. Lastly, we estimated the final model including all predictors with a *p*-value lower than 0.1 in at least one of the previous steps, as well as variables of clinical interest. The significance level was set to 0.05. Statistical analyses were conducted using SPSS 26.0 (IBM Corp., Armonk, NY) and R 4.1 [18], and Matlab 2020b (Mathworks Inc., Natick, MA) was used for graphics.

## Results

### Sample

**Fig. 1:**
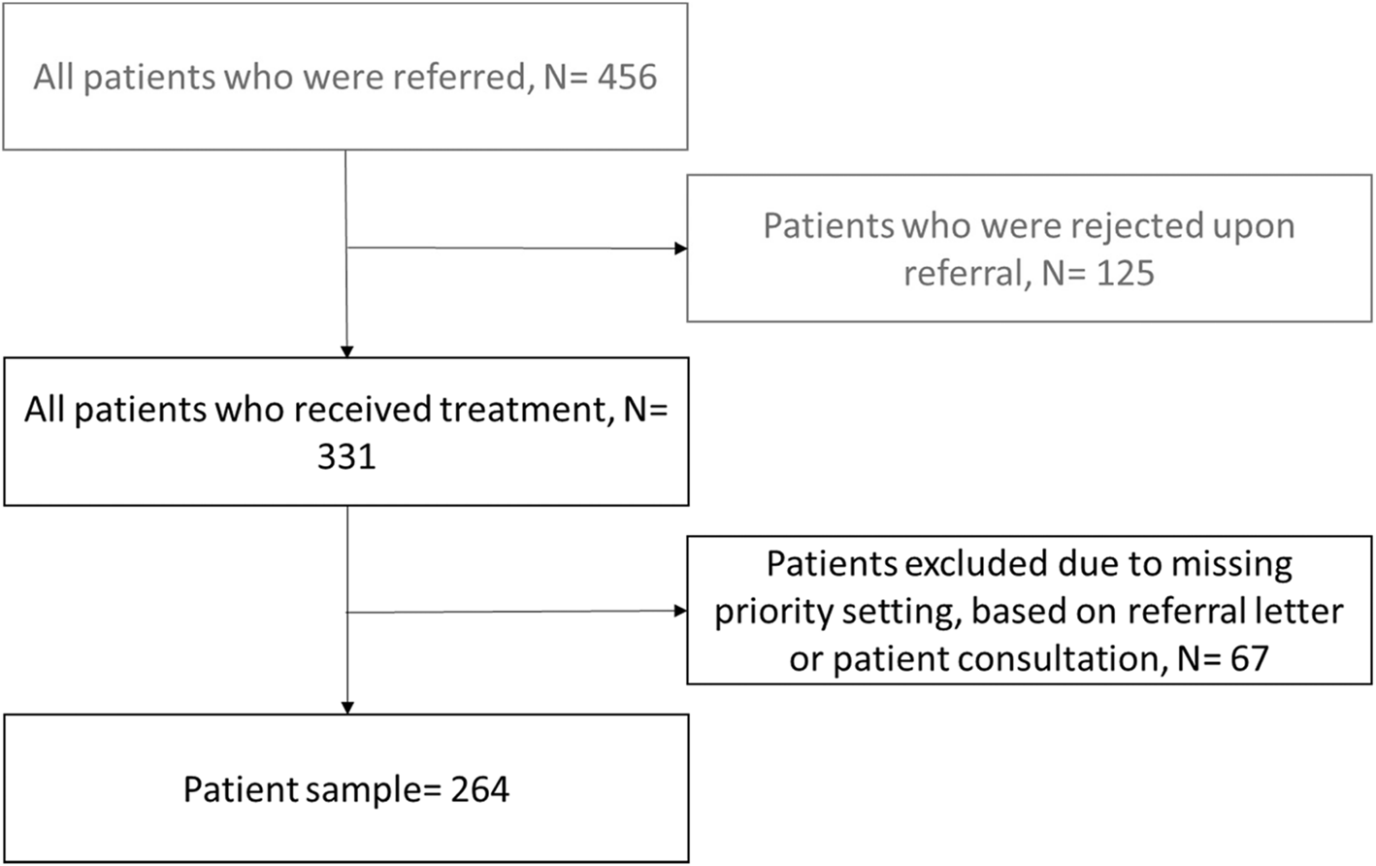
Flowchart of the inclusion process of the patient sample

In total, 331 referrals were initially included. Of these, 67 were excluded because of missing registration on one or both triages. The final patient sample consisted of 264 referrals (Fig. 1). Using one way ANOVAs (p < 0.05), we found no significant differences between the group of included versus excluded patients with regard to the variables shown in Table 1.

**Table 1:** Characteristics of the patient sample and system for triage decisions

Patient characteristics	Valid N	Value
Sum score QRef-MH^a^	264	8.2 (2.31)
Age^b^	264	
< 25 years		78 (30%)
26–45 years		135 (51%)
46–65 years		42 (16%)
> 65 years		9 (3%)
Sex: (Female)^b^	264	165 (63%)
Received specialised mental health care in the last 5 years^b^	260	114 (44%)
Registered diagnosis^b^	234	
Substance abuse		18 (8%)
Psychosis/schizophrenia		4 (2%)
Bipolar disorder		13 (6%)
Depression		38 (16%)
Anxiety/OCD		54 (23%)
Personality disorder		10 (4%)
ADHD		18 (8%)
Developmental disorder		5 (2%)
Other diagnosis		51 (22%)
Patients with more than one diagnosis		23 (10%)
System characteristics	Valid N	Value
Same assessor for both priorities^b,c^	264	93 (35%)
Specialist usually takes part in priority assessments^b^	263	179 (68%)
Specialist met the patient at the first consultation^b,d^	263	125 (48%)
Specialist knows the patient well^b^	263	70 (27%)
Time between priority assements (weeks)^a,c^	256	4.9 (3.0)

^a^ Mean (SD). ^b^ N(%). ^c^ Referral letter and patient consultation. ^d^ Priority setting based on second hand information from health care professional partaking in the interprofessional team: No

The maximum acceptable waiting time (triage decision) set on the basis of referral letter information varied from 1 to 26 weeks (mean: 10.4, SD: 5.09). When using information gained from patient consultation, the mean maximum acceptable waiting time was 9.7 weeks (SD: 5.34, min: 0, max: 30). Actual time between the two triage assessments were on average 4.9 weeks (SD: 3.0). There were more women (63%) than men (38%), 81% of patients were under 45 years of age and 39% suffered from anxiety or depression. Two thirds of the triage decisions based on consultation with the patient were conducted by specialists who regularly take part in priority setting. In over half of the cases (53%), hospital specialists assessed the maximum acceptable waiting time based on second-hand information from structured clinical interviews performed by other healthcare personnel from the interprofessional team (Table 1).

### Reliability of triage decisions

**Fig. 2:**
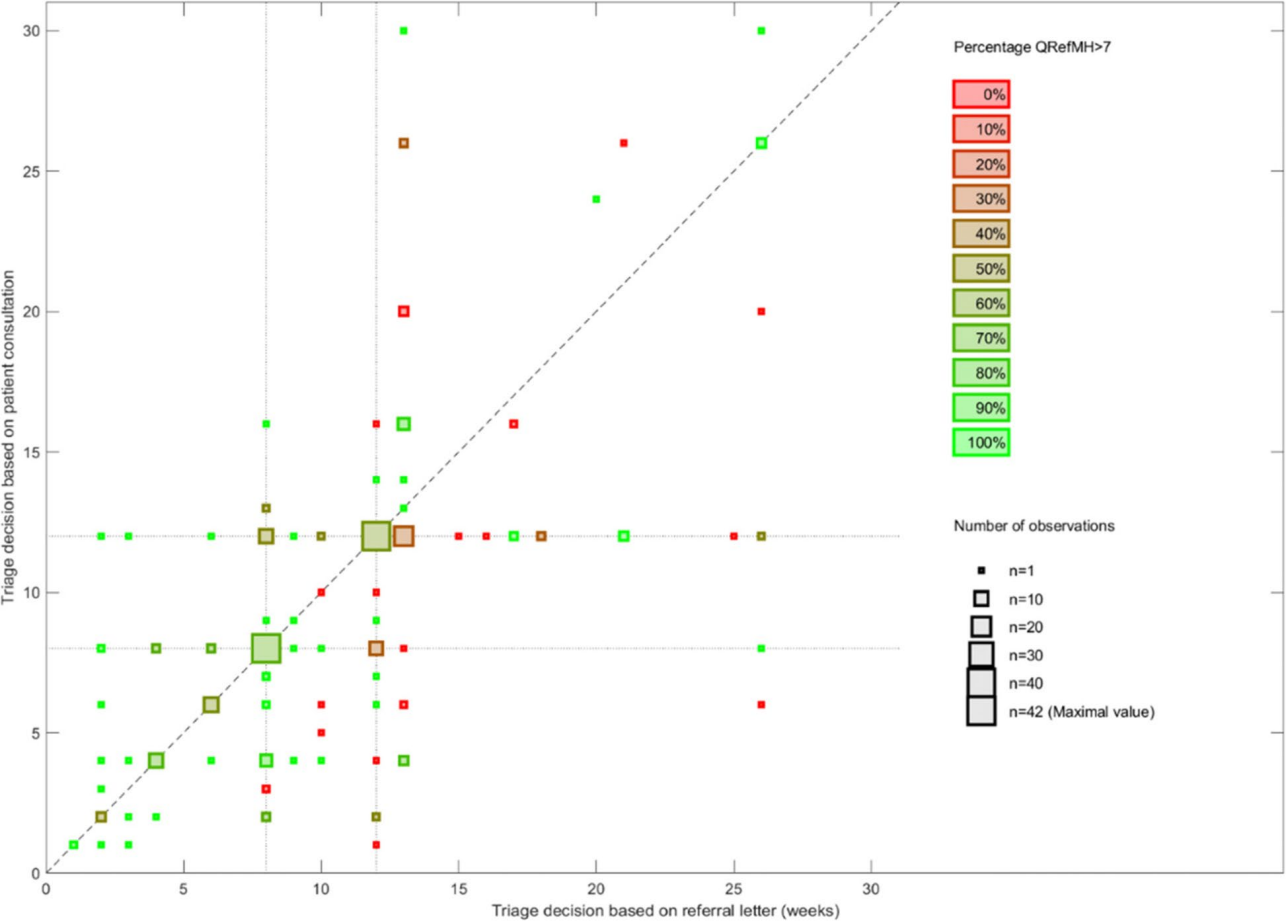
Maximum acceptable waiting time in weeks as assessed based on referral information (x-axis) and based on information from patient consultation (y-axis). The size of the plots indicates the number of observations. The colour indicates the percentage of high-quality referral letters from red (no referral letters with scores exceeding 7 on a 0–16 point scale) to green (the referral letter quality exceeds 7 in all cases)

In 54% of cases (*N* = 143), the triage decisions were reliable, meaning that the triaging based on the referral information was equal or similar to the triage decisions based on patient consultation. In addition, 27% of referrals were given a higher priority when using information from the patient consultation, indicating underestimation in the existing system in terms of how urgently patients were considered to need help. For the remaining 19% of patients, the existing system of referral assessment triaged the patient to a shorter acceptable waiting time compared with when referral was based on patient consultation (Table 2). Among the patients whose treatment needs were underestimated, 32 were triaged to a maximum acceptable waiting time of 5 weeks or less when seeing the specialist (Fig. 2). Most of these patients (*N* = 24) were triaged to 8 weeks or more when assessed on the basis of referral information.

**Table 2:** Descriptions of patient groups receiving either similar or different triage decisions. Overestimation means that a longer maximum acceptable waiting time was given when based on a patient consultation compared with that based on a referral letter. Underestimation means that a shorter maximum acceptable waiting time was given based on the patient consultation compared with the referral letter

		Deviation (weeks)		
Priority setting	N(%)	Mean(SD)	Minimum	Maximum
Similar	143 (54%)	0.2 (0.4)	-1	1
Overestimation	51 (19%)	4.9 (3.3)	1	17
Underestimation	70 (27%)	5.9 (3.7)	1	-20

### Impact of referral letter quality

**Fig. 3:**
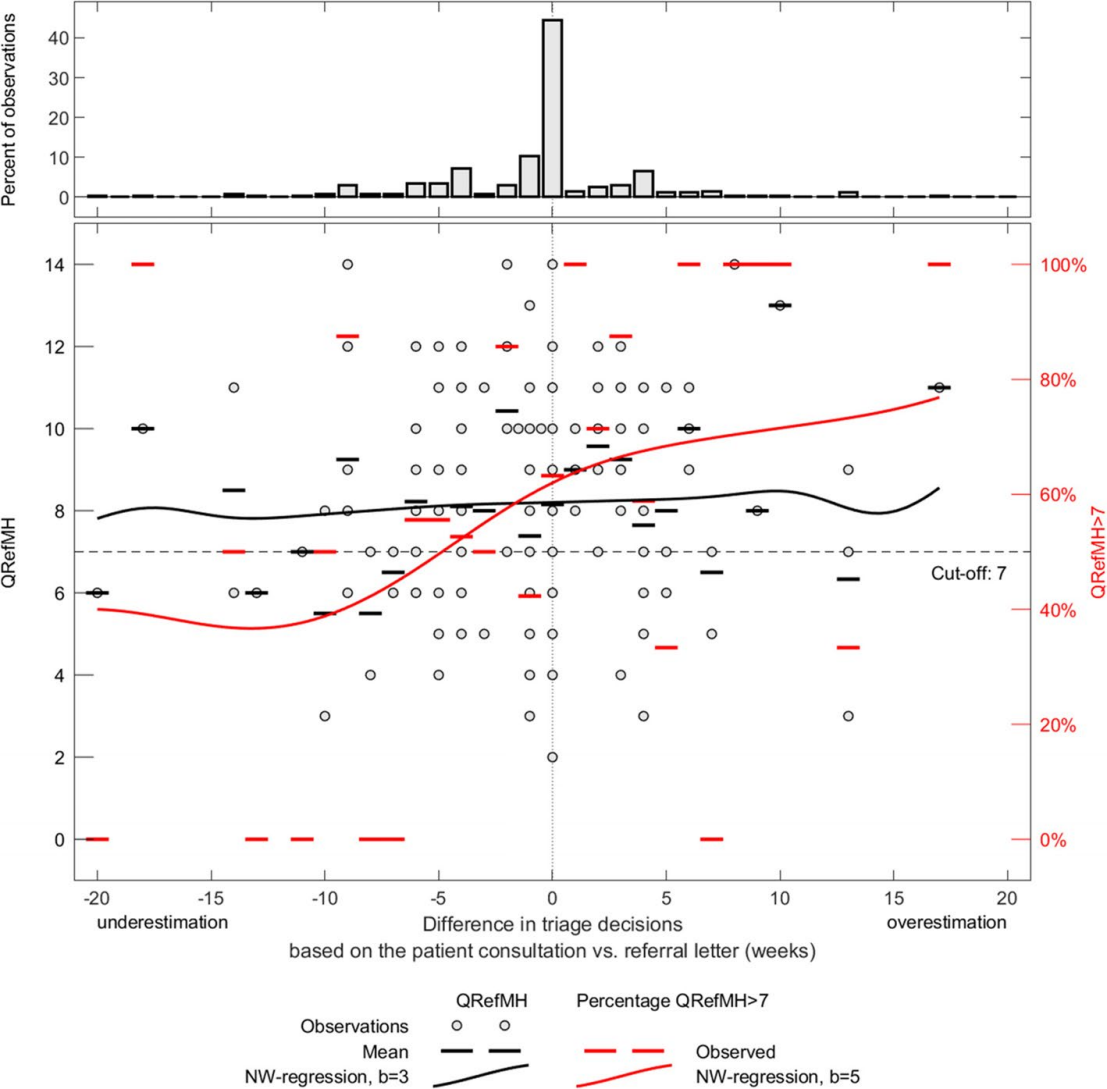
Percentage of referrals with a QRef-MH score between 0 and 7, or between 8 and 16, and assessments of maximum acceptable waiting time, either indicating that the patient received a priority that was too low (left-hand side) or too high (right-hand side). The mean difference in weeks (solid lines) and the ± 1 SD interval (dotted line) were estimated using a Nadaraya-Watson kernel estimator with a bandwidth of 5 and 3 weeks. Dots indicate observations

The mean QRef-MH score for the 264 referrals was 8.2 (SD: 2.31), rated on a scale from 0–16. The mean score was slightly lower in the group with reliable triage decisions (mean: 8.0; SD: 2.18), compared with the other group (mean: 8.4; SD: 2.45). The binary logistic regression analysis revealed that the effect of referral letter quality on reliability of triage decisions was not significant (Table 3). The Nadaraya-Watson kernel estimation suggests a lower proportion of high-quality referral letters (QRef-MH sum score > 7) among the underestimated referrals compared with the overestimated referrals. In the group of overestimated referrals, 60%–80% of the referrals were categorised as having high quality. Among the underestimated referrals, less than 50% were of high quality (Fig. 3).

**Table 3:** Binary logistic regression for the relationship between appropriate triage decisions and QRef-MH scores, demographic variables and system variables, presented as unadjusted, adjusted and final models

	Unadjusted model			Adjusted model		Final model	
				*N* = 251		*N* = 263	
	N	OR (95% CI)	*P*-value	OR (95% CI)	*P*-value	OR (95% CI)	*P*-value
QRef-MH score	264	0.93 (0.84–1.04)	.200	0.94 (0.84–1.06)	.324	0.94 (0.84–1.05)	.248
Age	264		.052		.083		.106
< 25 years	78	1		1		1	
26–45 years	135	0.51 (0.28–0.91)		0.48 (0.26–0.88)		0.54 (0.30–0.97)	
46–65 years	42	0.38 (0.17–0.81)		0.43 (0.19–0.98)		0.41 (0.18–0.91)	
> 65 years	9	0.63 (0.16–2.53)		0.76 (0.16–0.36)		0.59 (0.14–2.43)	
Sex (Female)	264	1.64 (1.0–2.72)	.052	1.72 (1.01–2.94)	.047	1.73 (1.03–2.92)	.039
Received help from specialised mental health care in the last 5 years	260	1.11 (.68–1.81)	.686	1.11 (0.65–1.87)	.711	-	-
Specialist usually takes part in priority assessments	263	1.80 (1.07–3.04)	.027	1.90 (1.12—3.4)	.031	1.74 (1.01–3.00)	.046
Time between priority assements (weeks)	256	1.02 (.94–1.10)	.711	1.02 (.93—1.11)	.638	-	-

### Impact of other factors

The binary logistic regression analysis revealed that the patient’s sex and the specialist’s experience in priority setting were significant predictors of the reliability of triage decisions in all models (Table 3). Female patients were more likely to be given a reliable triage, and experienced specialists had a higher rate of reliable triage decisions. The analysis indicated that the number of weeks to wait before the first consultation or having received specialised mental health care the last 5 years did not predict the reliability of triage decisions. The patient’s age group had a significant impact on the reliability of triage decisions. Patients between 26 and 65 years of age were less likely to receive a reliable assessment than patients under 25 years of age.

## Discussion

We found that the existing triage system for patients referred to specialist mental health care was reliable for more than half of the referrals we examined. In 46% of cases, however, we found indications of over- or underestimation of the urgency of need for specialist health care. The quality of referral letters was not found to explain the risk of over- and underestimation. However, we found indication of a greater proportion of low-quality referral letters in the subgroup of cases in which a meeting with the patient indicated a higher priority than the referral information.

The reliability of specialist mental health care’s triage based on referral information, and the risk of under- and overestimation, as found in the current study, have been debated for many years [9, 10]. Under- or overestimation of the urgency of patients’ need when decisions are based on referral information has been highlighted previously [9, 10] and measures to improve reliability of triage decisions have been implemented [11]. In a similar setting to the present study, Holman and colleagues reported low interrater reliability for priority setting between local mental health centres, using vignettes of referral letters [2]. The current results indicated that almost half of the patients were triaged differently when priority was based on meeting the patient compared with using referral information only, in accordance with the assumed risk described in previous literature.

To reduce the patient safety risk associated with the handover between primary and secondary care, improved structures have been suggested [9, 11]. Improving referral letters has been proposed as an important task, building on studies revealing the substantial potential for improved adherence to guidelines for recommended content [2, 9]. This approach is only partially supported by the current results. We found that the quality of referral letters on a continuous scale played a relatively minor role in explaining the reliability of priority setting. However, when we applied a cut-off value of 7 in QRef-MH scores of 0–16 to classify low vs high quality, referral letter quality appeared to have an impact. We found a higher proportion of low-quality referral letters in the sub-group in which the urgency of needs was underestimated compared with the group in which it was overestimated. The risk of suicidal behaviour and the negative impact on prognosis mean that underestimation of the acceptable waiting time constitutes a patient safety risk [4, 5]. Although improving the quality of referral letters to an acceptable level may reduce the risk of underestimation, we are not aware of other studies reporting the same finding.

### Strengths and limitations

Timely access to specialist health care is essential for high-quality care. A major strength of the present study is that it investigated the trustworthiness of one of the most commonly used methods for deciding if and when patients should receive specialised health care. We compared this with another method used by many mental health services, in which the needs of the patient are assessed by consultation. Using a naturalistic sample of patients and minimal intervention from researchers during the study period, we consider the results to represent the existing health services accurately. In addition, testing the hypothesis that referral letters’ quality can explain the reliability of triage decisions between patients is important for developing evidence-based quality improvement interventions.

However, several limitations of the current study should be considered. Although we have used the terms “overestimated” and “underestimated”, we cannot state which priority is correct. Triage decisions based on the referral letter information may be more or less correct than decisions based on meeting the patient, even though it is assumed that adding information from meeting the patient to the information from the referring doctor is likely to provide a better foundation for triage decisions. Our intention was to investigate the trustworthiness of the existing system, not to define “correct priority”. We have no information about the consequences for patients or the services for those categorised as under- or overestimated.

We adjusted for possible confounding factors, such as specialists’ experience with priority setting and whether or not the patient had received specialised mental health care in the last 5 years. However, other factors that we were not able to adjust for may have affected the results. For example, we did not know if or to what degree the patients’ health status changed or if they received other health care interventions while waiting for specialised mental health care. However, the number of weeks between first and second triage did not significantly impact the reliability of triage decisions. This indicates that alterations in the patients’ health status can explain the differences in the two decisions only to a limited degree.

Triages performed after meeting the patient are not a part of the current referral system. Factors affecting the way that the hospital specialist triages a patient after the first consultation may be different to the factors that influence the triage based on referral letter information, due to regulations and guidelines prompting the latter. It is unknown to what degree the grounds on which the triage decisions were made were similar, e.g. regarding whether or not the hospital specialist had met the patient himself at the second triage. The second triages was in some cases performed by the same mental health specialist or by a specialist with knowledge about the first triage decision. This potential bias may have reduced the differences between the two triages we found. Also, we did not include information as to which particular hospital specialists made the triage decisions, and could therefore not explore to what degree clustering within specialists affected the results. In addition, the accuracy of assessments of maximum acceptable waiting time would be expected to be affected by regulations in each country. In the Norwegian setting, triage decisions between referred patients are regulated by law. The generalisability to other patient groups and services is unknown. We believe that our findings provide insight into challenges faced in most two-tiered health services.

### Implications

The present study indicates the need for more reliable triage methods. Our results suggested that improving the quality of referral letters may be beneficial only when the quality score is lower than average. The complexity of prioritising between patients implies that a risk of inappropriate triage decisions may always be present, as found in the current study. Specialised mental health care services should encourage patients, next of kin and the referring doctor to ask for a second assessment if they believe the urgency of treatment need has been under-communicated or misunderstood.

In future studies, we recommend further investigation of the reliability of the existing referral assessment system in terms of treatment and patient outcomes, further exploration of the characteristics of patients or situations that involve a greater risk of under- or overestimation of the urgency of needs, and investigation of the reliability of referral rejection. Also, the focus of the present study has been the health system’s reliability of giving priority. We have not investigated alternative systems that may eliminate the present waiting time for first consultation or interventions to reduce the burden for patients that are waiting. We urge future research to do so.

## Conclusion

The current findings indicated that the referral system was sufficient for providing reliable triage decisions of patients in most cases. However, the risk of over- and underestimation was present, also among the patients having an urgent need for specialised mental health care. The limited association found between referral letters' quality and the reliability of triage indicate that the existing emphasis on improving referral letters may have a limited impact on ensuring timely access to specialist mental health care. Deciding the urgency of treatment need is complex. Confounding factors may have been overlooked, and our findings may not apply to other health service settings. Further research is needed to investigate the validity of our findings in other settings, and for developing interventions to improve the reliability of mental health triage in the referral process. Validation of the current findings for sub-groups of patients or situations in future studies is recommended.

## Data Availability

The datasets used and analysed during the current study are available from the corresponding author on reasonable request.
